# The crucial role of machine learning models in predicting current childhood asthma: model comparison, calibration, and SHAP-based interpretation

**DOI:** 10.3389/fpubh.2026.1823225

**Published:** 2026-07-23

**Authors:** Aditya Chakraborty, A. K. M. Raquibul Bashar

**Affiliations:** 1 Department of Epidemiology, Biostatistics and Environmental Health, Joint School of Public Health, Old Dominion University, Norfolk, VA, United States; 2 Department of Mathematics and Computer Science, Augustana College, Rock Island, IL, United States

**Keywords:** asthma predictive modeling, calibration plots, childhood asthma, early detection, machine learning models, model validation, SHAP

## Abstract

**Background:**

Asthma is one of the most prominent chronic diseases in children and one of the most challenging ailments to diagnose in infants and preschoolers in the United States. Predictive models can be instrumental in improving early diagnosis, personalized treatment strategies, and disease progression. By utilizing nationalized data, this study focuses on building and comparing high-performing analytical predictive models based on the relevant risk factors and identifying the most influential predictors.

**Methods:**

We analyzed cross-sectional BRFSS Asthma Call-Back Survey data (2011–2020; *N* = 9,813) and randomly split participants into training and testing sets. An XGBoost model (hyperparameters tuned via grid search) was developed and compared with SVM, random forest, LASSO, and GBM using accuracy, AUC, precision, and recall. Calibration was evaluated with reliability plots and improved using Platt scaling and isotonic regression. Predictor contributions were examined using variable-importance (VIP) and Shapley Additive Explanations (SHAP) plot.

**Results:**

Of the five predictive models, the XGBoost was found to be the best performing model with AUC: 0.95, followed by random forest (AUC: 0.9345), GBM (AUC: 0.9341), SVM (AUC 0.9304), and LASSO (AUC 0.88); however, the random forest model was found to have the highest sensitivity (0.9786), and hence preferred for initial screening of asthma. On the independent test set, calibration (10-bin reliability curves; Brier/ECE/intercept–slope) improved most with isotonic regression, specifically for Random Forest (ECE 0.0158 to 0.0086; intercept −0.174 to −0.010), whereas Platt scaling often worsened calibration, with AUC remaining largely stable across models (AUC ≈ 0.92–0.95). The top two contributing predictors were overnight hospitalization visits and time since the last asthma medication, accounting for 24.62 and 20.92%, respectively, of the asthma status, from the VIP.

**Conclusion:**

The analytical methodology of model development was found to be instrumental in the discovery of behavioral health-risk knowledge and to visualize the significance of predictive modeling from a multidimensional behavioral health survey. These insights can be instrumental in predicting different types of chronic lung diseases affecting people of all ages and can be useful for clinicians to diagnose asthma at an early stage, allowing for early intervention and proactive management.

## 1 Introduction

Asthma is one of the most common major non-communicable diseases and continues to impact on the quality of life. Globally, asthma is ranked 16th among the leading causes of years lived with disability and 28th among the leading causes of burden of disease, as measured by disability-adjusted life years. Around 300 million people have asthma worldwide, and it is likely that by 2025, a further 100 million may be affected with this disease [31]. Despite progress in understanding asthma’s pathophysiology, the condition continues to pose a major public health challenge, with significant implications for individual and societal welfare [25]. Asthma is the most prominent chronic disease in children and one of the most challenging ailments to diagnose in infants and preschoolers. Castro-Rodriguez, Jose A., explains the usefulness of the Asthma Predictive Index (API) to address the recurrent wheezing of preschoolers/infants at the school-age [18]. There are multiple risk factors (deficiency in vitamin D, tobacco smoke exposure, air pollution, maternal anxiety or depression, allergen exposure, genetic risk factors, etc.) that have been found to be associated with asthma, and the differences in distributions of these risk factors can be attributed to the differences in prevalence that has a declining pattern in some parts of the world [58]. Although asthma is often manageable through inhaled medications, its clinical course varies, with some individuals experiencing persistent or recurrent symptoms into adulthood [43]. Consequently, identifying predictive factors and constructing models to forecast asthma development in children is crucial, potentially facilitating early intervention and personalized management strategies.

Machine learning techniques have become promising tools for predicting and managing various health conditions, including asthma, owing to their capacity to analyze complex datasets and discern intricate patterns not readily apparent via traditional statistical methods [33, 23]. Leveraging large datasets, machine learning algorithms can identify risk factors, predict disease onset, and personalize treatment approaches, potentially improving patient outcomes and reducing healthcare costs [14]. The Behavioral Risk Factor Surveillance System serves as a valuable data source for studying health-related behaviors and risk factors among the US population [28]. Applying machine learning techniques to BRFSS data can yield valuable insights into predicting childhood asthma, potentially leading to more effective prevention and management strategies [16]. In recent years, researchers are prone to using sophisticated data-driven machine learning and decision-making models in applied sciences because of their high predictive power and learning abilities from data [7, 8, 10, 13, 24, 21, 59, 22, 20, 57, 19, 9]. Chakraborty and Tsokos [23] developed a predictive model for pancreatic cancer data using the XGBoost algorithm (eXtreme Gradient Boosting) and validated the model’s performance by comparing it with ten deep neural network (DNN) models [54, 26] grown sequentially with different activation functions and optimizers [52, 1]. Daines et al. studied seven existing prediction models to support the diagnosis of asthma in children and adults in primary care and found that patients who reported wheezing, variability of symptoms, and a history of allergy or allergic rhinitis were associated with asthma [30]. Colicino, Silva, et al. [29] used validated tools for predicting childhood asthma based on MEDLINE and EMBASE (1946–2017) data for all available childhood asthma prediction models, and recommended the inclusion of larger cohorts, and more predictors into the model, along with more advanced statistical techniques to obtain better predictive accuracy. The primary goal of this study was to develop and compare machine-learning models to predict current childhood asthma in the context of the need for accurate, interpretable, and clinically reliable asthma risk prediction tools based on national ACBS data. The specific goals were to: (1) compare the discrimination and classification performance of five supervised learning models; (2) check for the reliability of predicted probabilities by using reliability curves and calibration metrics; (3) explore whether reliability can be improved by post-hoc Platt scaling or isotonic regression of the predicted probabilities; and (4) identify influential predictors using variable-importance and SHAP based explainability methods.

## 2 Materials and methods

### 2.1 Data

Data used in this study were acquired from the CDC (Centers for Disease Control and Prevention) website https://www.cdc.gov/brfss/acbs/index.htm. For the purpose of the study, we visited the survey data and documentation module of the BRFSS database. Then we selected the Asthma Return Visit Survey (ACBS) sub-database and downloaded the 2011 through 2020 Child Return Visit Survey Data. The nature of the study design was cross-sectional. During the study, data were collected from 9,813 children aged 1 to 18 years from the BRFSS Asthma Callback Survey (ACBS) system [66, 65]. Call-back survey data were collected on all states in the US and its territories. So, the developed predictive models can be generalized to the US children (Ages: 1–18) population. Since the data was publicly available with de-identified information, we were not required to gain any privacy consent.

Prior data preprocessing was done before the development of models. Invalid responses such as ‘do not know,’ refusal and unavailable responses were examined following the ACBS codebook. When the number of participants with missing or unknown responses was high for categorical predictors, those responses were included as explicit categories to prevent excluding individuals with incomplete responses to a survey. No synthetic oversampling technique, like SMOTE was used because the distribution of outcomes was moderately balanced (current asthma: 6,442/9,813; 65.6%, no current asthma: 3,371/9,813; 34.4%). Rather, stratified data partitioning and model evaluation through sensitivity, specificity, AUC and confusion matrix were utilized to track possible over-fitting to the majority class.

### 2.2 Outcome variable

The outcome variable for the study “Curr_ASTH_fct” (Label: Still Have Asthma Combined BRFSS and Call Back) was selected as the outcome variable to determine whether the children still had asthma during the time of the call-back interview. Cross-sectional data [64] were analyzed in 9813 participants, including 6,442 (65.6%) with current asthma status positive, and 3,371 (34.4%) with current asthma negative from the BRFSS ACBS system.

### 2.3 Predictors

After acquiring data from the CDC website, 28 predictors were selected as the initial list of risk factors based on the data codebook and questionnaires available on the website, as mentioned earlier. The Least Absolute Shrinkage and Selection Operator (LASSO) regression [35, 56] was used to select the final list of variables/risk factors to be included in the model-building process. Based on this statistical variable selection technique, 25 variables were selected, where all risk covariates were categorical except Age. This variable was treated as an ordinal variable with 5 categories. The predictor variables included in this study are listed in the Table 1.

**Table 1:** List of variables included in the prediction model building scheme.

Variables	Variable type	Variable names	Variable description
Response Variable	Categorical	Curr_ASTH_fct	Does the individual still have asthma?
Predictor Variables	Categorical	GENDER	Gender of the individual
		TIME_LAST_DIAG	Time Since Diagnosis
		LAST_MED	How long has it been since the individual took asthma medication
		LAST_SYMTMS	How long has it been since the individual last had any symptoms of asthma?
		SYMTMS_DAYS	During the past 30 days, on how many days did the individual have any symptoms of asthma?
		SYMTMS_ALTIME	Does the individual have symptoms all the time? “All the time” means symptoms that continue throughout the day.
		Asthma Episode	During the past 12 months, has the individual had an episode of asthma or an asthma attack?
		Attack Duration	How long did the most recent asthma episode or attack last?
		HEALTH_INSURANCE	Does the individual have any type of health insurance/coverage
		INSURANCE_TYPE	Type of insurance coverage
		INSURANCE_GAP	During the past 12 months, was there any time that the individual did not have any health insurance or coverage?
		FLU_SHOT	During the past 12 months, did the individual have a flu shot?
		FLU_SPRAY	During the past 12 months, did the individual have a flu vaccine that was sprayed in [his/her] nose?
		USUAL_ACTIVITY	During the past 30 days, was the daily activity limited a little, a moderate amount, or a lot due to asthma?
		HOSP_VISIT	Overnight Hospital stay due to asthma in last 12 months
		AIR_CLEANER	Is an air cleaner or purifier regularly used inside the individual’s home?
		GAS_COOK	Is gas used for cooking in the individual’s home?
		MOLD	In the past 30 days, has anyone seen or smelled mold or a musty odor inside the individual’s home?
		Indoor Pets	Does the individual’s home have pets such as dogs, cats, hamsters, birds, or other feathered or furry pets that spend time indoors?
		Bedroom Pets	Is the pet allowed in the bedroom?
		MATTRESS_COVER	Does the individual use a mattress cover that is made especially for controlling dust mites?
		CARPET/RUG	Does the individual have carpeting or rugs in [his/her] bedroom?
		DYCARE_PET	Are there any pets such as dogs, cats, hamsters, birds, or other feathered or furry pets in the individual’s room at daycare?
		DYCARE_MOLD	Is there a mold problem at the daycare?
	Ordinal	Age	Age groups

### 2.4 Machine learning models

The machine learning models used in this study are briefly summarized as follows:

GBM: Gradient Boosting Machine (GBM) is a powerful machine learning technique that combines predictions from multiple decision trees to generate final predictions. This ensemble method is commonly used for both classification and regression problems, as itcan effectively model complex, nonlinear relationships in data. GBMs work by iteratively building a sequence of decision trees, where each new tree focuses on correcting the errors of the previous one. This iterative process leads to an ensemble that is more accurate and robust than any single tree alone [49]. GBMs have been widely adopted across various industries and domains due to their ability to handle a wide range of data types and deliver strong predictive performance. The core principle behind GBMs is to construct new base learners that are highly correlated with the negative gradient of the loss function associated with the entire ensemble. This allows the algorithm to concentrate on areas where the existing ensemble performs poorly, gradually improving the overall predictive power. GBMs offer flexibility in terms of the loss function, as researchers can choose from a variety of loss functions or even implement their task-specific loss function, making the algorithm highly customizable to any particular data-driven task [37, 39].

XGBOOST: One of the most popular and widely used GBM algorithms is extreme gradient boosting (XGBoost). In the field of machine learning, one algorithm that has attracted considerable attention is XGBoost, a highly efficient and scalable implementation of the gradient-boosting technique. XGBoost has been widely acclaimed for its outstanding performance in numerous machine learning and data mining competitions, securing its position as the go-to choice for ensemble methods [12, 23, 46]. Furthermore, the thorough examination of the parameter tuning process in XGBoost can be invaluable to researchers across different fields. The parameter tuning process can offer insights into how various parameter configurations impact the algorithm’s performance, empowering researchers to make well-informed choices when optimizing XGBoost for their specific data and modeling needs [47]. By gaining a deeper understanding of the nuances in parameter setting, researchers can harness XGBoost’s flexibility to achieve exceptional predictive accuracy and efficiency within their respective domains.

LASSO: The Least Absolute Shrinkage and Selection Operator (LASSO) is a statistical technique primarily used for feature selection and data model regularization.

In contrast to traditional regression models, LASSO has the unique capability to shrink certain regression coefficients to zero, effectively performing feature selection [61, 63]. This characteristic makes LASSO a valuable tool for predictive modeling, as it enables researchers to identify the most influential variables that drive the prediction of the target variable. The academic literature has extensively documented the utility of the LASSO algorithm in predictive modeling [2, 42]. LASSO proves particularly advantageous when working with high-dimensional datasets, where the number of predictor variables exceeds the number of observations. In such scenarios, the LASSO method can help prevent overfitting by selecting only the most relevant features, leading to more robust and generalizable predictive models. This feature selection ability makes LASSO a valuable tool for developing accurate and parsimonious predictive models, especially in fields with complex, high-dimensional data. Furthermore, multicollinearity, a prevalent issue in regression analysis where predictor variables are highly correlated, can be addressed with LASSO. LASSO can successfully reduce the impacts of multicollinearity by reducing some regression coefficients to zero, producing models that are more stable and comprehensible.

Random Forest: The random forest (RF) technique is a powerful and widespread machine learning approach that combines the outputs of multiple decision trees to generate a single predictive outcome. This ensemble method is particularly advantageous for both classification and regression tasks, as the aggregation of multiple models enhances the overall accuracy and robustness of the predictions [15]. The algorithm operates by creating a collection of individual decision trees, each trained on a random subset of the training data and a random subset of the features. The final prediction is then derived by aggregating the outputs of all the individual trees, either through majority vote or averaging. This ensemble strategy helps to mitigate the risk of overfitting and provides a more stable and reliable model compared to a single decision tree [62]. One of the key strengths of the random forest algorithm is its ability to handle large datasets with a high number of features [6]. Unlike traditional regression methods, the random forest algorithm can automatically detect and handle nonlinear relationships in the data, making it well-suited for complex and real-world problems. Additionally, the algorithm can accommodate a variety of data types, including numerical, categorical, and textual data, and is robust to outliers and missing values [3]. Furthermore, the random forest algorithm provides a measure of variable importance, which can be useful for feature selection and understanding the relative contribution of different variables to the model’s predictions [36]. This information can assist researchers and practitioners in identifying the most crucial factors driving the model’s outputs, which can be valuable for both interpretability and model optimization.

SVM: Support Vector Machines (SVM) are a powerful supervised machine learning algorithm widely used for classification and regression tasks [17, 55]. These models aim to identify an optimal hyperplane that maximally separates data points belonging to different classes, effectively acting as a decision boundary for classifying new, unseen instances. Furthermore, support vector machines are renowned for their computational efficiency, as the optimization problem they seek to solve is convex in nature and can beaddressed using well-established algorithmic techniques [50]. A key advantage of SVMs is their ability to generalize well, even with relatively small training datasets, making them particularly useful for domains where data collection is challenging or expensive. Additionally, SVMs have demonstrated effectiveness in handling high-dimensional data and complex non-linear relationships by leveraging kernel functions, which transform the input data into a higher-dimensional space where linear separation becomes feasible [38, 55].

Deep-learning architectures were not included because the study used a moderately sized, structured tabular survey dataset composed primarily of categorical predictors rather than high-dimensional imaging, waveform, or free-text data. Also, deep learning models are often considered “black-box” models because their complex internal architecture can make it difficult to directly interpret how specific input features influence predictions. For this data structure, tree-based ensemble models and regularized linear models are generally more sample-efficient, easier to tune, and more interpretable than deep neural networks, reducing the risk of overfitting while preserving clinical interpretability.

### 2.5 Model building, validation, and calibration

To develop and evaluate the predictive models, the dataset was randomly partitioned into a training set (60%) and a test set (40%). All stages of the model development were performed independently for the predictive algorithms described under section 2.3. Model development and tuning were performed exclusively on the training dataset to avoid overfitting and enhance the performance of the models. To optimize the performance of each algorithm, a grid search with cross-validation was employed using the training dataset [11, 23]. For the LASSO model, regularization techniques were implemented to improve feature selection and mitigate overfitting. The ensemble methods (XGBoost, RF, and GBM) were fine-tuned to achieve a balance between model complexity and generalization capability. For the Support Vector Machine model, various kernel functions were tested to maximize classification accuracy. The predictive performance of each trained model was evaluated using an independent test set. Four key metrics were employed: sensitivity, specificity, area under the receiver operating characteristic (ROC) curve, and overall accuracy. Sensitivity and specificity were used to evaluate the model’s ability to accurately classify positive and negative instances, respectively. The AUC was used to determine the model’s discriminative capacity across various thresholds, while accuracy was used to summarize the proportion of correctly classified instances within the test dataset. By evaluating these diverse metrics, a comprehensive assessment of model performance was warranted, particularly in balancing the trade-offs between false positives and false negatives. The model that produced the highest AUC and balanced sensitivity and specificity was considered the most effective for prediction in this study.

Additionally, we evaluated and optimized the calibration of predicted probabilities of the binary outcome. Once the individual base models had been fitted to the training set, we measured calibration on a separate test set with reliability (calibration) curves composed of 10 quantile-based bins, with the average predicted probability in each bin compared to the observed rate of event occurrence. Calibration was also measured by Brier score, expected calibration error (ECE), and calibration intercept and slope [41, 53]. In order to have a more calibrated set of probabilities, we used two post-hoc probability calibration methods on a held-out calibration set: Platt scaling (sigmoid calibration) and isotonic regression [5]. Isotonic regression is a monotonic recalibration (non-parametric) algorithm that best approximates a piecewise-constant function between the raw model scores and probabilities, flexible to correct systematic under- or overestimation, but preserving the rank of the order. The test set was then used to compare the quality of probability prior to and subsequent to calibration.

## 3 Results

**Figure 1:**
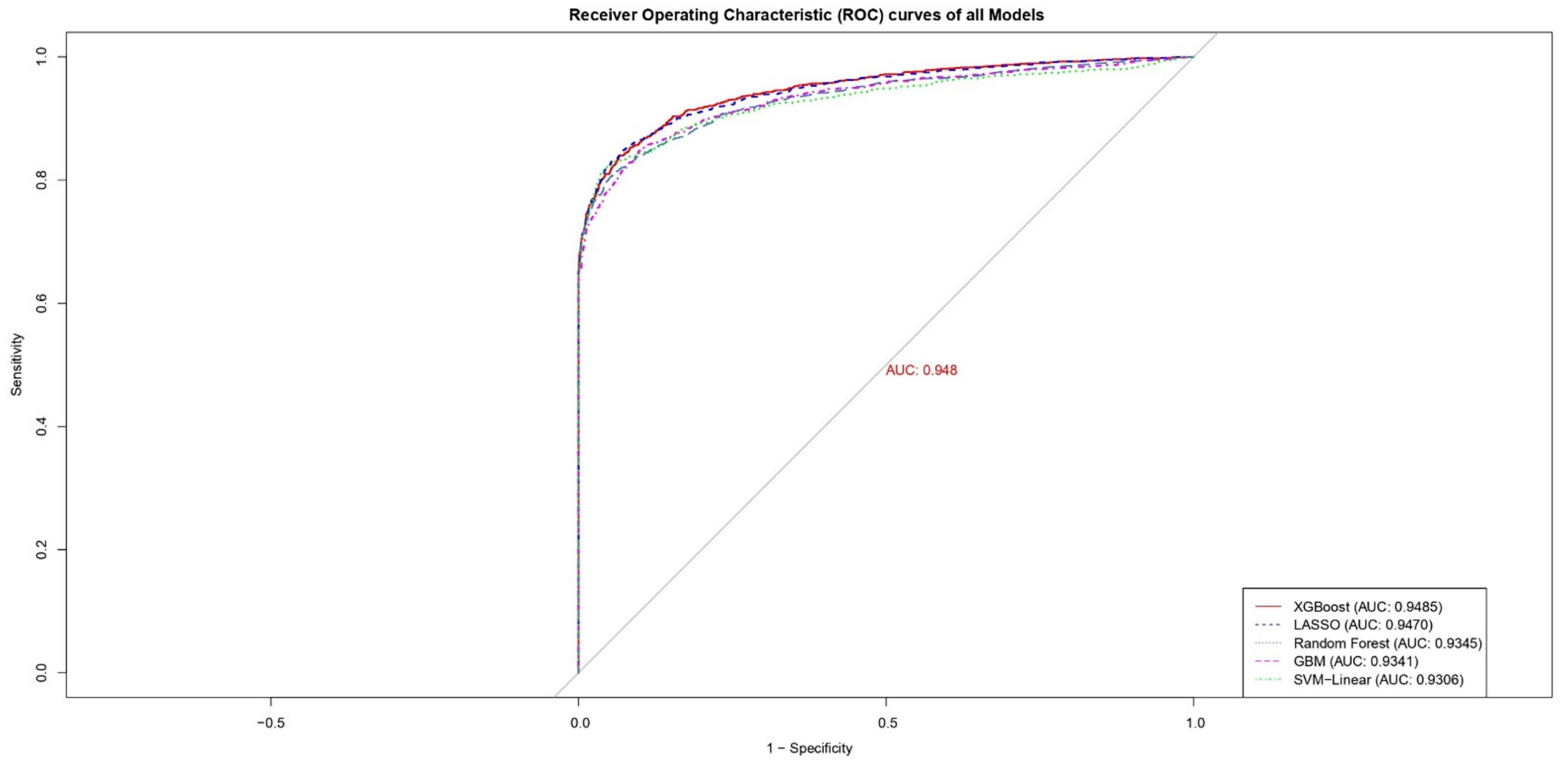
Model comparison via ROC Curves.

In this section we describe different performance matrices based on the five machine learning (ML) models. Table 2 summarizes the performance of five machine learning models—XGBOOST, LASSO, SVM, Random Forest (RF), and Gradient Boosting Machine (GBM), in predicting childhood asthma, evaluated using four metrics: accuracy, specificity, sensitivity, and area under the curve (AUC). As the table illustrates, XGBOOST demonstrated the best overall predictive accuracy (90.67%, AUC: 94.85%) indicating strong discriminative ability and reliable identification of true asthma cases, followed by LASSO (90.59%), SVM (90.34%), RF (90.16%), and GBM (88.99%). Also, XGBOOST showed the highest AUC value, followed by LASSO, RF, GBM, and SVM. The prediction accuracy and performance were found to be high, consistent, and robust for all five models (Table 2 and Figure 1). The random forest model was found to have the highest sensitivity (97.86%) followed by the XGBOOST (96.55%), and LASSO (96.47%). GBM provided a balanced trade-off between sensitivity (93.21%), specificity (80.93%), and AUC (93.41%), making it a reliable alternative.

**Table 2:** Comparing different ML models via four performance matrices.

Models	Accuracy	Specificity	Sensitivity	AUC
XGBOOST	0.9067	0.7945	0.9655	0.9485
LASSO	0.9059	0.7938	0.9647	0.9470
SVM	0.9034	0.8854	0.9119	0.9306
RF	0.9016	0.7545	0.9786	0.9345
GBM	0.8899	0.8093	0.9321	0.9341

The values of the comparison matrix are presented as shown in the following Table 2.

Table 3 shows confusion matrix showing that all five models correctly identified a large number of children with current asthma, with Random Forest producing the fewest false negatives (*n* = 40) and the highest true positives (*n* = 1,810), supporting its strong sensitivity for screening. XGBoost and LASSO showed balanced performance with relatively low false negatives and moderate false positives.

**Table 3:** Confusion matrix of the ML models on the independent test set.

Model	True positive	False negative	True negative	False positive
XGBOOST	1,786	64	761	197
LASSO	1,785	65	760	198
SVM	1,687	163	848	110
RF	1,810	40	723	235
GBM	1,724	126	775	183

Counts were calculated on the independent test set using the final classification threshold. Positive class = current asthma (Curr_ASTH_fct = Yes). False negatives indicate children with current asthma classified as no current asthma; false positives indicate children without current asthma classified as current asthma.

### 3.1 Model calibration

The analytic dataset was partitioned into three mutually exclusive subsets to facilitate unbiased calibration of probabilities and evaluation: a training set (*n* = 4,914; 52.5%) to fit each individual base classifier, a separate calibration set (*n* = 1,638; 17.5%) to only learn post-hoc probability recalibration mappings, and finally an independent test set (*n* = 2,808; 30.0%) to only evaluate performances. A stratified sampling technique was used to divide the sample into subsets to maintain the prevalence of Curr_ASTH_fct.

**Figure 2:**
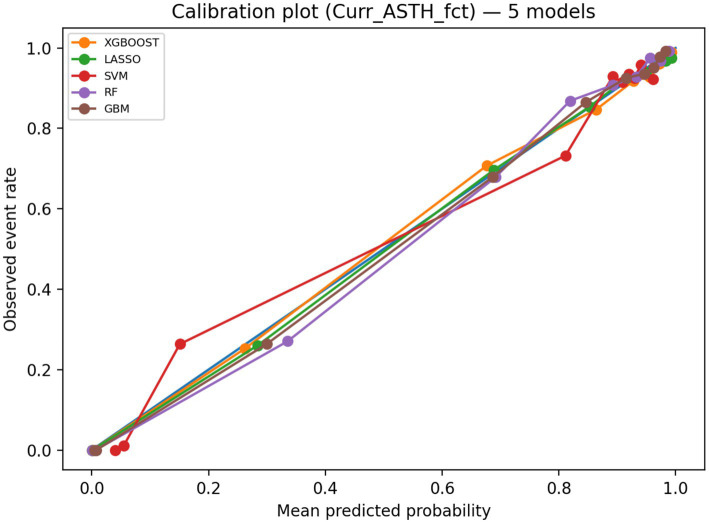
Calibration of model-reported probabilities (base models).

**Figure 3:**
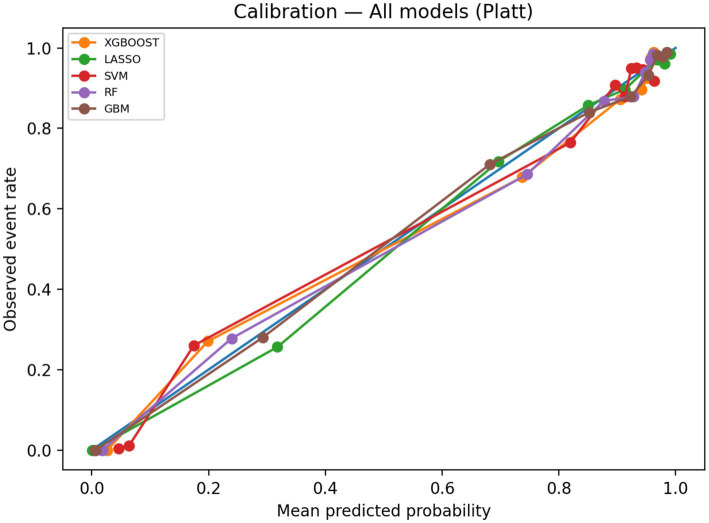
Calibration after Platt scaling.

**Figure 4:**
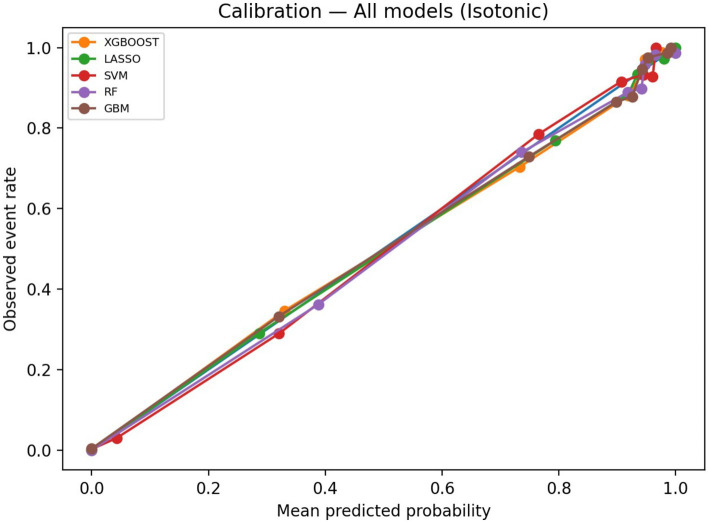
Calibration after isotonic regression.

Across the five classifiers, the calibration probabilities for the outcome Curr_ASTH_fct was evaluated on the independent test set (*n* = 2,808) using reliability curves (10 quantile bins) and quantitative metrics (Brier score, ECE, and calibration intercept/slope) and then improved using post-hoc Platt scaling and isotonic regression fitted on the held-out calibration set (*n* = 1,638). Generally, Platt scaling failed to enhance calibration and often deteriorated probability agreement (greater ECE and more negative intercepts); however, isotonic regression was steady toward the calibration gains, especially with regard to the Random Forest model. Precisely, XGBoost was highly discriminating, with little threshold miscalibration (AUC 0.950; intercept −0.028; slope 0.973) and a smaller improvement of ECE with isotonic recalibration (ECE 0.0145 to 0.0123), whereas Platt scaling deteriorated calibration (ECE 0.0238; intercept −0.125). LASSO had been already well calibrated before recalibration (intercept −0.004; slope 0.951; Brier 0.0710), and Platt moved the probabilities again toward overprediction (intercept −0.151). SVM made the weakest probability estimates at baseline (Brier 0.0798; intercept 0.057; slope 0.936) with isotonic decreasing Brier (0.0773) but no decrease in ECE, and Platt increasing ECE (0.0263). Random Forest had the highest error of the baseline calibration (intercept −0.174; ECE 0.0158), and isotonic regression was effective for the model, which significantly improved calibration (ECE 0.0086) and the systematic bias (intercept −0.010) with the slope approaching the optimal value of 1 (0.942), whereas the gain was not significant for Platt. GBM, conversely, showed relatively good baseline calibration (ECE 0.0111; slope 1.048) and failed to increase with either recalibration technique. These findings suggest that discrimination was high. and pretty consistent across the models (Table 4). Figure 2 illustrates Calibration plots of all five ML models before any post-hoc calibration was applied. Figure 3 and Figure 4 illustrate the post-hoc calibration via Platt scaling and isotonic regression, respectively. Table 4 shows the calibration performance of the ML models for predicting the asthma outcome before and after post-hoc probability calibration on test data.

**Table 4:** Calibration performance of the ML models for predicting Curr_ASTH_fct before and after post-hoc probability calibration on test data.

Model	Calibration	N	AUC	Brier score	ECE (10 bins)	Calibration intercept	Calibration slope
XGBOOST	Uncalibrated	2,808	0.950	0.071	0.015	−0.028	0.973
XGBOOST	Platt	2,808	0.950	0.073	0.024	−0.125	1.009
XGBOOST	Isotonic	2,808	0.948	0.072	0.012	−0.031	0.952
LASSO	Uncalibrated	2,808	0.946	0.071	0.016	−0.004	0.951
LASSO	Platt	2,808	0.946	0.071	0.015	−0.151	1.035
LASSO	Isotonic	2,808	0.945	0.071	0.013	−0.042	0.946
SVM	Uncalibrated	2,808	0.920	0.080	0.016	0.057	0.936
SVM	Platt	2,808	0.920	0.080	0.026	−0.103	0.992
SVM	Isotonic	2,808	0.919	0.077	0.018	−0.034	0.931
RF	Uncalibrated	2,808	0.948	0.072	0.016	−0.174	1.154
RF	Platt	2,808	0.948	0.073	0.019	−0.165	1.056
RF	Isotonic	2,808	0.947	0.073	0.009	−0.010	0.942
GBM	Uncalibrated	2,808	0.948	0.073	0.011	−0.084	1.048
GBM	Platt	2,808	0.948	0.073	0.014	−0.094	1.015
GBM	Isotonic	2,808	0.947	0.073	0.014	0.068	0.896

### 3.2 Feature importance

Analyzing the main features influencing the performance of a predictive model is a critical component of statistical modeling. This task is especially vital in clinical settings, where comprehending the decision-making rationale of machine learning models is essential for making informed decisions [45, 60]. One approach to addressing this challenge is the use of SHAP (Shapley Additive exPlanations) values, which offer a comprehensive measure of feature importance [44]. Shapley values, which originate from game theory, provide a distinctive viewpoint on feature importance by evaluating the marginal contribution of each feature to the overall model performance [51]. This framework facilitates the identification of the key factors driving model predictions, enabling researchers and practitioners to gain a deeper understanding of the underlying mechanisms and potentially address issues such as overfitting or heterogeneity.

**Figure 5:**
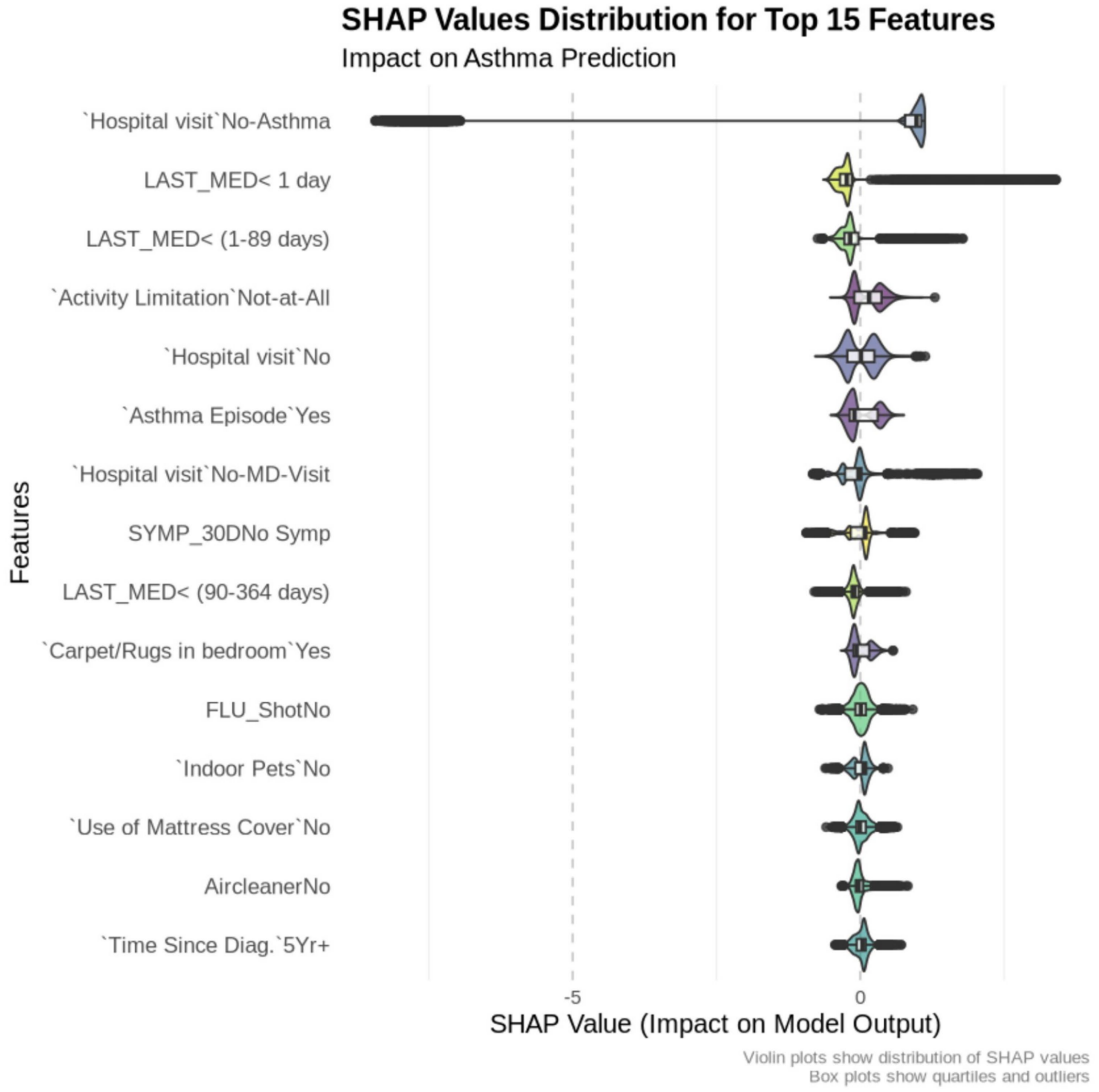
Shapley Plot for feature importance.

**Figure 6:**
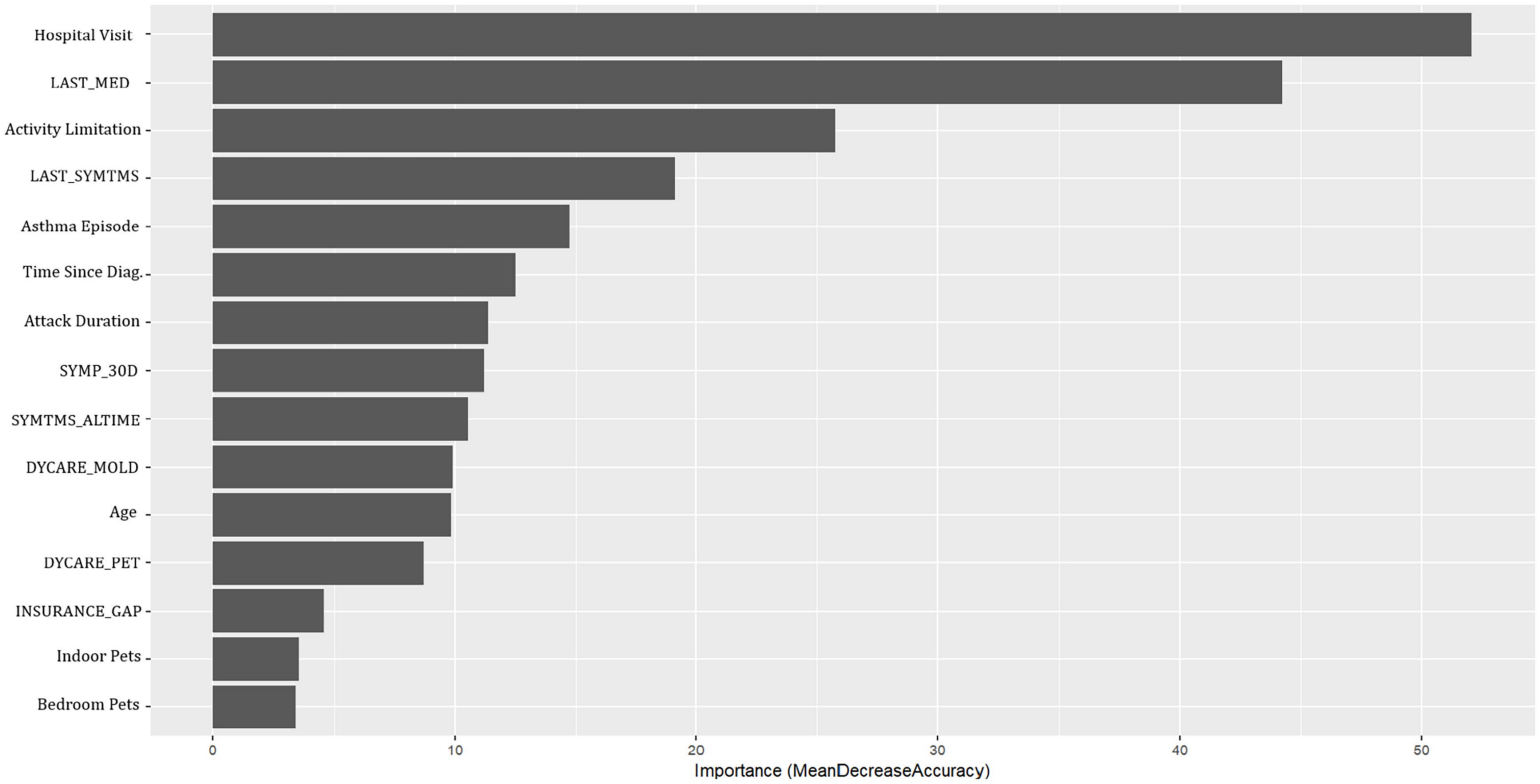
Top fifteen important features based on the random forest model.

The SHAP plot in Figure 5 helps to explain the associations of the features with the asthma status in terms of SHAP values. In the SHAP plot, the SHAP values are used to analyze the strength of the association between individual feature contributions and model predictions. This helps to interpret how much a particular feature influences the model’s decision-making. A high SHAP value for a specific feature indicates a strong relationship with the model’s output, implying that changes in that feature can significantly impact model predictions. From the SHAP plot, we see that not overnight hospitalization (hospital visit No-Asthma) has a strong negative impact on the asthma status (reduces the likelihood of asthma prediction). When a patient has no asthma-related hospital.

visits, it strongly decreases the likelihood of an asthma diagnosis. The narrow violin plot suggests this effect is consistent. Time since the last asthma medication less than 1 day (LAST MED < 1 day) and time since the last asthma medication less than 3 months (LAST MED < (1–89 days)) had a notable positive impact on asthma prediction. Activity limitation has a slightly negative impact on the outcome; patients reporting no activity limitations (Activity Limitation Not-at-All) reduced the predicted probability of asthma. No hospital visit in general has a negative impact. A recent asthma episode (Asthma Episode Yes) is strongly associated with asthma prediction. No MD visit (No-MD-Visit) has been found to have a dominating positive impact on the predicted outcome. The positive association suggests a higher risk of asthma-related complications when hospital visits do not involve an MD consultation. A negative association with the predicted asthma outcome was noted for the individuals with no asthma symptoms (SYMP 30D) in the last 30 days. Less recent medication use (LAST MED < (90–364 days)) was found to have a negative influence on asthma prediction, suggesting that medication use within the past 3 to 12 months might decrease the likelihood of asthma prediction. Some of the environmental factors (carpet/rugs in the bedroom, indoor pets, and air cleaner) were found to be significantly associated with asthma prediction. Not having the flu shot was found to be moderately associated with the predicted likelihood. Being diagnosed with asthma more than 5 years ago (time since the diagnosis 5 yr.+) has a slightly negative association with the asthma prediction, which is evident from the distribution of the SHAP values (leans toward the left side of zero). Figure 6 illustrates the variable importance plot (VIP) based on the random forest model.

Table 5 shows the top fifteen contributing predictors arranged according to the importance score and % of contribution to childhood asthma, which is also evident from Figure 6. The top four contributing predictors, according to the random forest (RF) algorithm, are overnight hospitalization visits, time since the last asthma medication.

**Table 5:** % Contribution of the top fifteen predictors to the RF model.

Predictors	Importance	% Contribution
Hospital Visit	52.10	21.56
LAST_MED	44.24	18.32
Activity Limitation	25.78	10.67
LAST_SYMTMS	19.11	7.91
Asthma Episode	14.73	6.10
Time Since Diag.	12.51	5.18
ATTACK/EPI_DUR	11.38	4.71
SYMP_30D	11.22	4.64
SYMTMS_ALTIME	10.54	4.36
DYCARE_MOLD	9.90	4.10
Age	9.82	4.07
DYCARE_PET	8.70	3.60
INSURANCE_GAP	4.57	1.90
Indoor Pets	3.53	1.46
Bedroom Pets	3.40	1.41

used, hindrance of usual activity due to asthma, and time since the last asthma symptom, accounting for 24.62, 20.92, 12.19, and 9.03%, respectively, to the outcome.

## 4 Discussion

Asthma is a chronic respiratory condition with significant public health implications due to its prevalence, associated healthcare costs, and impact on quality of life. In this study, five different predictive models were compared based on four different matrices. The XGBOOST emerged as the best-performing model overall, demonstrating the highest predictive accuracy (0.9067), sensitivity (0.9655), and AUC (0.9485) (Table 2). These metrics reflect the model’s strong ability to correctly classify both asthma and non-asthma cases, with particularly high effectiveness in detecting true positives. Where accuracy can be misleading for unbalanced data, AUC deals with the class imbalance by evaluating the model’s ability to discriminate between classes across all possible thresholds [34]. Although the study data was not imbalanced (asthma yes = 65.65%, & asthma no = 34.35%), the AUC metric shows a consistent pattern regarding the models’ predictive power, and robustness to correctly discriminate the outcome categories.

In the context of childhood asthma prediction, a high level of predictive performance is critical, as it ensures that most children at risk of developing asthma are accurately identified while minimizing misclassification. Accurate and early detection of children at risk of asthma can inform prompt interventions, such as environmental adjustments, early pharmacological intervention, and enhanced monitoring. The high AUC further validates the model’s capability to differentiate between affected and unaffected children, thereby supporting its application as a reliable tool for decision-making within pediatric primary care settings or public health screening initiatives.

The Random Forest (RF) model exhibited the greatest sensitivity among the competing models. High sensitivity is extremely crucial in the early detection of asthma, as failure to identify affected individuals can lead to treatment delays, increased incidence of complications, and a worsening quality of life. This early diagnosis is vital for initiating early interventions, which can prevent disease progression and improve patient outcomes. High sensitivity also lessens the likelihood of false negatives by reducing the misdiagnosis of patients. Missing an asthma diagnosis can lead to inadequate treatment, exacerbation, and potentially severe health consequences. The ensemble-based architecture of the RF algorithm facilitates the identification of nuanced patterns and interactions among various risk factors, thereby enhancing its ability to detect potential asthma cases, even in their developing stages or when symptoms are unclear or ambiguous. However, the enhanced sensitivity of the RF model was associated with a reduction in specificity compared to the other models, potentially resulting in a higher rate of false-positive diagnoses. Despite this drawback, the prioritization of sensitivity is often warranted in the context of a screening instrument, particularly when applied to susceptible pediatric populations. Although initial over-identification may precipitate further evaluations, it ensures a minimal number of missed true cases, which constitutes an acceptable compromise given that early diagnosis can substantially improve long-term prognoses.

As far as implementation is concerned, these models would fit best as decision support models and not as standalone diagnostic models. One possible deployment strategy would be the incorporation of the final model within an EHR-based or mobile health workflow that automatically accesses commonly collected asthma history, medication use, symptom severity, health care utilization, and information on environmental exposure. The output should be in the form of a calibrated risk probability, with an understandable explanation of the key factors in the prediction, so that the clinician can focus on follow-up, remind the child’s family of the asthma action plan, and recognize children who are at a higher risk who may need more monitoring. Thresholds may be adjusted to match purpose of use; higher thresholds are desirable for population screening and more balanced thresholds for clinical decision support.

As demonstrated in Table 2, the ML models revealed high sensitivity, and this means that they are powerful to identify the individuals who are at high risk of poor asthma outcomes. Nonetheless, the values of specificity were slightly lower, which represent a possible trade-off between identifying the true positives correctly and reducing the false positives. This in the real world of screening applications would mean that occasionally people can be wrongly identified as high risk and that further clinical evaluation or preventative measures might be unwarranted. However, in most cases, greater sensitivity should be a priority in public health surveillance, where the cost of false negatives is usually greater than the cost of false positives. The model threshold might be calibrated by future applications to find a balance between sensitivity and specificity, depending on the purpose of the model, e.g., screening, clinical decision support, or population risk stratification.

Important information about the factors affecting asthma diagnosis and severity was described using SHAP values. The model holds significant potential for informing public health interventions and policymaking due to its excellent predictive accuracy and interpretability. The SHAP analysis emphasizes how healthcare utilization, such as hospital stays and use of recent medication, are significant predictors of asthma, reflecting the severity of the condition and management strategies. These results are consistent with previous research showing that frequent hospital stays and ER visits are indicators of poorly managed asthma [32, 48]. By incorporating the model into clinical decision-making processes, high-risk individuals may be identified early, allowing for rapid therapies to lessen the consequences associated with asthma. Asthma predictions are also found to be significantly influenced by environmental factors, including indoor dogs, carpets, and the absence of air purifiers, highlighting the importance of allergens exposure in asthma treatment. These findings highlight the necessity of environmental health initiatives aimed at lowering exposure to indoor allergens [27]. For example, especially in homes with asthmatic members, public health initiatives could encourage allergen-reducing practices, including minimizing pet exposure, eliminating carpets, and utilizing HEPA filters [40].

Asthma predictive models have significant usefulness for several reasons. These predictive models can be useful in curating personalized treatment plans and hence improve the quality of life of the patients diagnosed with asthma. By predicting the course of asthma for individual patients, healthcare providers can tailor treatments to meet specific needs, improving outcomes and reducing unessential interventions, thus helping patients manage their condition better, leading to an enhanced quality of life. Effective asthma management guided by efficient predictive models can reduce hospital admissions and emergency visits, significantly lowering healthcare costs. Finally, these models can inform public health strategies and policies by identifying potential disease trends, contributing risk factors, and the impact of environmental or genetic factors on asthma prevalence and outcomes.

### 4.1 Limitation

One of the major limitations of this study is the lack of quantitative information available for building machine-learning models. Most of the variables in the survey questionnaire that we considered for model building were categorical in nature. While most ML algorithms perform better on quantitative features, including quantitative information in the development phase of the models might help in increasing the accuracy and relative performance. Also, our models were inherently limited by the features available in the BRFSS data. Because the BRFSS data is cross-sectional, causality could not be determined between the contributing features and the outcome. Another constraint is that the self-reported BRFSS data may be influenced by social desirability and recall bias, potentially impacting the accuracy of our predictive models. Despite these limitations, a larger number of observations and Machine learning models such as XGBoost can potentially provide insight to comprehend the complex nonlinear relationship between potential attributes and the targeted outcome.

The data provided in the BRFSS Asthma Call-Back Survey (ACBS) is self-reported and thus prone to recall and social desirability biases, and hence undermines the accuracy of major variables, including asthma attack duration, adherence to medication, and hospitalization visits. Categorical response options can also conceal within-group variation as well as decrease measurement accuracy. All these can result in nondifferentiation misclassification and weaken true associations. The ACBS, however, is a national representative, standardized surveillance system, and future research that incorporates objective data sources (e.g., claims or clinical records) may continue to confirm these results. Because the ACBS data are cross-sectional, the temporal ordering between predictors and current asthma status could not be established. Therefore, the findings should be interpreted as predictive associations rather than evidence of causal relationships.

Finally, the present study did not include external validation using an independent dataset. Although ACBS provides national U. S. surveillance data, model performance may vary across healthcare systems, international populations, pediatric practice settings, and groups with different access to asthma care or environmental exposures. Future validation should evaluate transportability in later ACBS waves, other national data sources such as NHIS, and clinical EHR datasets. Such validation would help determine whether the predictors and calibrated probabilities remain stable across populations and settings [4].

## 5 Conclusion

The findings of the study suggest that machine learning models, specifically XGBOOST and random forest, hold considerable promises for improving early asthma diagnosis, facilitating prompt interventions, and enhancing health outcomes for children at risk. Future research should concentrate on validating these models in external cohorts, exploring integration into electronic health records, and assessing their real-world clinical utility. Moreover, future studies may be reinforced with better quantitative and longitudinal data sources to improve the model intensity and causality. Connecting the data of the BRFSS Asthma Call-Back Survey (ACBS) to the data of electronic health records or environmental exposure (e.g., spirometry measurements, allergen status) would enable a more accurate description of disease progression. Longitudinal follow-up would also allow evaluation of time dynamics, i.e., changes in asthma control, medication compliance, and exposure patterns, thus enhancing the prediction of exacerbation risk at a later stage. The incorporation of predictive algorithms into routine pediatric care has the potential to facilitate personalized risk assessment and reduce the burden of childhood asthma through proactive management.

## Data Availability

The original contributions presented in the study are included in the article/supplementary material, further inquiries can be directed to the corresponding author/s.

## References

[1] Agostinelli F Hoffman M Sadowski P Baldi P. Learning activation functions to improve deep neural networks. arXiv preprint arXiv:14126830. (2014). doi: 10.48550/arXiv.1412.6830

[2] Ahmadieh-Yazdi A Mahdavinezhad A Tapak L Nouri F Taherkhani A Afshar S. Using machine learning approach for screening metastatic biomarkers in colorectal cancer and predictive modeling with experimental validation. Sci Rep. (2023) 13:19426. doi: 10.1038/s41598-023-46633-8, 37940644 PMC10632378

[3] Ao Y Li H Zhu L Ali S Yang Z. The linear random forest algorithm and its advantages in machine learning assisted logging regression modeling. J Pet Sci Eng. (2019) 174:776–89. doi: 10.1016/j.petrol.2018.11.067

[4] Bang KM Hnizdo E Doney B. Prevalence of asthma by industry in the us population: a study of 2001 nhis data. Am J Ind Med. (2005) 47:500–8. doi: 10.1002/ajim.20170, 15898089

[5] Barlow RE Brunk HD. The isotonic regression problem and its dual. J Am Stat Assoc. (1972) 67:140–7.

[6] Barren˜ada L Dhiman P Timmerman D Boulesteix A-L Van Calster B. Understanding overfitting in random forest for probability estimation: a visualization and simulation study. Diagn Progn Res. (2024) 8:14. doi: 10.1186/s41512-024-00177-1 39334348 PMC11437774

[7] Bashar AKMR Goudarzi M Tsokos CP. A machine learning classification model for detecting prediabetes. J Data Anal Inf Process. (2024) 12:462–78. doi: 10.4236/jdaip.2024.123024

[8] Chakraborty A Nicklett E. J. Understanding Drivers of Physical Activity Through Explainable Machine Learning: The Role of Disability and Social Determinants. J Phys Act Health. (2026) (published online ahead of print 2026). doi: 10.1123/jpah.2026-0347 42413907

[9] AKM RaquibulBashar and Chris PTsokos. Statistical parametric analysis on democracy data. Open Access Libr J. (2019) 6:1–18. doi: 10.4236/oalib.1105828

[10] Bashar AKMR Tsokos CP. Parametric and statistical analysis on corruption perception index (CPI) of countries of the world based on world governance index (WGI) data of transparency international (TI). Open J Soc Sci. (2024) 12:396–416. doi: 10.4236/jss.2024.129023

[11] Belete DM Huchaiah MD. Grid search in hyperparameter optimization of machine learning models for prediction of HIV/AIDS test results. Int J Comput Appl. (2022) 44:875–86. doi: 10.1080/1206212X.2021.1974663

[12] Belida MJCM Begum A David SA Kannan E Senthil K Naveena NR. "Predictive modeling for medical insurance malpractice using random forest and xgboost". In: 2024 International Conference on Communication, Computing and Internet of Things (IC3IoT). Chennai, India: IEEE (2024). p. 1–5.

[13] Belsher BE Smolenski DJ Pruitt LD Bush NE Beech EH Workman DE . Prediction models for suicide attempts and deaths: a systematic review and simulation. JAMA Psychiatry. (2019) 76:642–51. doi: 10.1001/jamapsychiatry.2019.0174, 30865249

[14] TanBhowmik R. A multi-modal respiratory disease exacerbation prediction technique based on a spatio-temporal machine learning architecture. arXiv preprint arXiv:210303086. (2021). doi: 10.48550/arXiv.2103.03086

[15] Biau G’e Scornet E. A random forest guided tour. TEST. (2016) 25:197–227. doi: 10.1007/s11749-016-0481-7

[16] Budhathoki N Bhandari R Bashyal S Lee C. Predicting asthma using imbalanced data modeling techniques: evidence from 2019 Michigan brfss data. PLoS One. (2023) 18:e0295427. doi: 10.1371/journal.pone.0295427, 38060576 PMC10703315

[17] Campbell C Ying Y. "Learning with support vector machines". In: Synthesis Lectures on Artificial Intelligence and Machine Learning. San Rafael, CA: Morgan & Claypool Publishers (2011)

[18] Castro-Rodriguez JA. The asthma predictive index: a very useful tool for predicting asthma in young children. J Allergy Clin Immunol. (2010) 126:212–6. doi: 10.1016/j.jaci.2010.06.032, 20624655

[19] Chakraborty A Tsokos CP. A real data-driven clustering approach for countries based on happiness score. Amfiteatru Economic. (2021) 23:1031–1045. doi: 10.24818/EA/2021/S15/1031

[20] Chakraborty A Tsokos CP. A modern approach of survival analysis of patients with pancreatic cancer. Am J Cancer Res. (2021) 11:4725. 34765290 PMC8569348

[21] Chakraborty A Tsokos CP. A real data-driven analytical model to predict happiness. Sch J Phys Math Stat. (2021) 3:45–61. doi: 10.36347/sjpms.2021.v08i03.001

[22] Chakraborty A Tsokos CP. Survival analysis for pancreatic cancer patients using cox-proportional hazard (CPH) model. Glob J Med Res. (2021) 21:29–46. doi: 10.34257/gjmrfvol21is3pg29

[23] Chakraborty A Tsokos CP. An ai-driven predictive model for pancreatic cancer patients using extreme gradient boosting. J Statistical Theory Applications. (2023) 22:262–82. doi: 10.1007/s44199-023-00063-7, 30311153

[24] Chakraborty A Tsokos CP. Parametric and non-parametric survival analysis of patients with acute myeloid leukemia (aml). Open J Appl Sci. (2021) 11:126–48. doi: 10.4236/ojapps.2021.111009

[25] Chang W-C Livneh H Chen W-J Hsieh C-C Wang Y-H Lu M-C . Adding Chinese herbal medicine to routine care is associated with a lower risk of rheumatoid arthritis among patients with asthma: a population-based retrospective cohort study. Front Pharmacol. (2022) 13:895717. doi: 10.3389/fphar.2022.895717, 36059972 PMC9431954

[26] Cichy RM Kaiser D. Deep neural networks as scientific models. Trends Cogn Sci. (2019) 23:305–17. 30795896 10.1016/j.tics.2019.01.009

[27] Cipriani F Calamelli E Ricci G. Allergen avoidance in allergic asthma. Front Pediatr. (2017) 5:103. doi: 10.3389/fped.2017.00103, 28540285 PMC5423906

[28] Clark NM. Community-based approaches to controlling childhood asthma. Annu Rev Public Health. (2012) 33:193–208. doi: 10.1146/annurev-publhealth-031811-124532 22224877

[29] Colicino S Munblit D Minelli C Custovic A Cullinan P. Validation of childhood asthma predictive tools: a systematic review. Clin Exp Allergy. (2019) 49:410–8. doi: 10.1111/cea.13336, 30657220

[30] Daines L McLean S Buelo A Lewis S Sheikh A Pinnock H. Systematic review of clinical prediction models to support the diagnosis of asthma in primary care. NPJ Primary Care Respiratory Medicine. (2019) 29:19. doi: 10.1038/s41533-019-0132-z, 29777106 PMC5959853

[31] Dharmage SC Perret JL Custovic A. Epidemiology of asthma in children and adults. Front Pediatr. (2019) 7:246. doi: 10.3389/fped.2019.00246, 31275909 PMC6591438

[32] Dougherty RH Fahy JV. Acute exacerbations of asthma: epidemiology, biology and the exacerbation-prone phenotype. Clin Exp Allergy. (2009) 39:193–202. doi: 10.1111/j.1365-2222.2008.03157.x, 19187331 PMC2730743

[33] Ekpo RH Osamor VC Azeta AA Ikeakanam E Amos BO. Machine learning classification approach for asthma prediction models in children. Health Technol. (2023) 13:1–10. doi: 10.1007/s12553-023-00732-8

[34] ElSeddawy AI Karim FK Hussein AM Khafaga DS. Predictive analysis of diabetes-risk with class imbalance. Comput Intell Neurosci. (2022):3078025. doi: 10.1155/2022/3078025 36268149 PMC9578843

[35] Fan L Chen S Li Q Zhu Z. 2015 4th International Conference on Computer Science and Network Technology (ICCSNT) 1.IEEE (2015). p. 579–83.

[36] Gregorutti B Michel B Saint-Pierre P. Correlation and variable importance in random forests. Stat Comput. (2017) 27:659–78. doi: 10.1007/s11222-016-9646-1

[37] Kim C Park T. Predicting determinants of lifelong learning intention using gradient boosting machine (gbm) with grid search. Sustainability. (2022) 14:5256. doi: 10.3390/su14095256

[38] Kim J-H Kwon O-Y Hwang U-J Jung S-H Gwak G-T. Prediction model of subacromial pain syndrome in assembly workers using shoulder range of motion and muscle strength based on support vector machine. Ergonomics. (2024) 67:1237–46. doi: 10.1080/00140139.2023.2290983, 38039103

[39] Konstantinov AV Utkin LV. Interpretable machine learning with an ensemble of gradient boosting machines. Knowl Based Syst. (2021) 222:106993. doi: 10.1016/j.knosys.2021.106993

[40] Leas BF D’Anci KE Apter AJ Bryant-Stephens T Lynch MP Kaczmarek JL . Effectiveness of indoor allergen reduction in asthma management: a systematic review. J Allergy Clin Immunol. (2018) 141:1854–69. doi: 10.1016/j.jaci.2018.02.001, 29452202

[41] Lee G Kim W Oh H Youn BD Kim NH. Review of statistical model calibration and validation—from the perspective of uncertainty structures. Struct Multidiscip Optim. (2019) 60:1619–44. doi: 10.1007/s00158-019-02270-2

[42] Liang C Wanling L Maofeng W. Lasso-derived model for the prediction of bleeding in aspirin users. Sci Rep. (2024) 14:12507. doi: 10.1038/s41598-024-63437-6, 38822153 PMC11143346

[43] Locksley RM. Asthma and allergic inflammation. Cell. (2010) 140:777–83. doi: 10.1016/j.cell.2010.03.004, 20303868 PMC3134388

[44] Louhichi M Nesmaoui R Mbarek M Lazaar M. Shapley values for explaining the black box nature of machine learning model clustering. Procedia Comput Sci. (2023) 220:806–11. doi: 10.1016/j.procs.2023.03.107

[45] Lu S-C Swisher CL Chung C Jaffray D Sidey-Gibbons C. On the importance of interpretable machine learning predictions to inform clinical decision making in oncology. Front Oncol. (2023) 13:1129380. doi: 10.3389/fonc.2023.1129380, 36925929 PMC10013157

[46] McCarty D Lee D Park Y Kim HW. Exploring road safety through urban fabric characteristics and theory-driven prediction modeling with SEM-XGBoost. Environ Plan B Urban Anal City Sci. (2025) 52:303–21. doi: 10.1177/23998083241259069

[47] Mehdary A Chehri A Jakimi A Saadane R. Hyperparameter optimization with genetic algorithms and xgboost: a step forward in smart grid fraud detection. Sensors. (2024) 24:1230. doi: 10.3390/s24041230, 38400385 PMC10892895

[48] Minkovitz CS Andrews JS Serwint JR. Rehospitalization of children with asthma. Arch Pediatr Adolesc Med. (1999) 153:727–30. doi: 10.1001/archpedi.153.7.727, 10401806

[49] Natekin A Knoll A. Gradient boosting machines, a tutorial. Front Neurorobot. (2013) 7:21. doi: 10.3389/fnbot.2013.00021, 24409142 PMC3885826

[50] Noble WS. What is a support vector machine? Nat Biotechnol. (2006) 24:1565–7. doi: 10.1038/nbt1206-1565, 17160063

[51] Nohara Y Matsumoto K Soejima H Nakashima N. Explanation of machine learning models using shapley additive explanation and application for real data in hospital. Comput Methods Prog Biomed. (2022) 214:106584. doi: 10.1016/j.cmpb.2021.106584, 34942412

[52] Nwankpa C Ijomah W Gachagan A Marshall S. Activation functions: comparison of trends in practice and research for deep learning. arXiv preprint arXiv:181103378. (2018). doi: 10.48550/arXiv.1811.03378

[53] Pavlovic M. Understanding model calibration–a gentle introduction and visual exploration of calibration and the expected calibration error (ECE). arXiv preprint arXiv:250119047. (2025). doi: 10.48550/arXiv.2501.19047

[54] Rayavarapu K Krishna KKV. "Prediction of cervical cancer using voting and dnn classifiers". In: 2018 International Conference on Current Trends towards Converging Technologies (ICCTCT) IEEE (2018). p. 1–5.

[55] Salcedo-Sanz S Rojo-Álvarez J’e L Mart’ınez-Ramón M Camps-Valls G. Support vector machines in engineering: an overview. Wiley Interdiscip Rev Data Min Knowl Discov. (2014) 4:234–67. doi: 10.1002/widm.1125

[56] Shaon MSH Karim T Shakil MS Hasan MZ. A comparative study of machine learning models with lasso and shap feature selection for breast cancer prediction. Healthc Anal. (2024) 6:100353. doi: 10.1016/j.health.2024.100353

[57] Shipe ME Deppen SA Farjah F Grogan EL. Developing prediction models for clinical use using logistic regression: an overview. J Thorac Dis. (2019) 11:S574–84. doi: 10.21037/jtd.2019.01.25, 31032076 PMC6465431

[58] Stern J Pier J Litonjua AA. Asthma epidemiology and risk factors. Semin Immunopathol. (2020). 42:5–15. doi: 10.1007/s00281-020-00785-1 32020334

[59] Stiglic G Kocbek P Fijacko N Zitnik M Verbert K Cilar L. Interpretability of machine learning-based prediction models in healthcare. Wiley Interdiscip Rev Data Min Knowl Discov. (2020) 10:e1379. doi: 10.1002/widm.1379

[60] Hué S Hurlin C Pérignon C Saurin S. Measuring the driving forces of predictive performance: application to credit scoring. Management Science. (2026) 1–17. doi: 10.1287/mnsc.2023.02025

[61] Tibshirani R. Regression shrinkage and selection via the lasso. J R Stat Soc B Stat Methodol. (1996) 58:267–88.

[62] Lo Vercio L Amador K Bannister JJ Crites S Gutierrez A MacDonald ME . Supervised machine learning tools: a tutorial for clinicians. J Neural Eng. (2020) 17:062001. doi: 10.1088/1741-2552/abbff2, 33036008

[63] Wang J Xu Y Liu L Wu W Shen C Huang H . Comparison of lasso and random forest models for predicting the risk of premature coronary artery disease. BMC Med Inform Decis Mak. (2023) 23:297. doi: 10.1186/s12911-023-02407-w, 38124036 PMC10734117

[64] Wilkins R Schiffmacher S Gatewood A Conway L Greiner B Hartwell M. Asthma medications in schools: a cross-sectional analysis of the asthma call-back survey 2017-2018. J Osteopath Med. (2017) 122:581–6. doi: 10.1515/jom-2022-0063, 35918304

[65] Winer RA Qin X Harrington T Moorman J Zahran H. Asthma incidence among children and adults: findings from the behavioral risk factor surveillance system asthma call-back survey—United States, 2006–2008. J Asthma. (2006) 49:16–22. doi: 10.3109/02770903.2011.637594, 22236442

[66] Zahran HS Bailey CM Qin X Moorman JE. Assessing asthma control and associated risk factors among persons with current asthma–findings from the child and adult asthma call-back survey. J Asthma. (2015) 52:318–26. doi: 10.3109/02770903.2014.956894, 25144551 PMC4552346

